# PENGUINN: Precise Exploration of Nuclear G-Quadruplexes Using Interpretable Neural Networks

**DOI:** 10.3389/fgene.2020.568546

**Published:** 2020-10-27

**Authors:** Eva Klimentova, Jakub Polacek, Petr Simecek, Panagiotis Alexiou

**Affiliations:** ^1^Faculty of Informatics, Masaryk University, Brno, Czechia; ^2^Central European Institute of Technology (CEITEC), Masaryk University, Brno, Czechia

**Keywords:** bioinformatics and computational biology, machine learning, deep neural network, G quadruplex, web application, genomic, imbalanced data classification

## Abstract

G-quadruplexes (G4s) are a class of stable structural nucleic acid secondary structures that are known to play a role in a wide spectrum of genomic functions, such as DNA replication and transcription. The classical understanding of G4 structure points to four variable length guanine strands joined by variable length nucleotide stretches. Experiments using G4 immunoprecipitation and sequencing experiments have produced a high number of highly probable G4 forming genomic sequences. The expense and technical difficulty of experimental techniques highlights the need for computational approaches of G4 identification. Here, we present PENGUINN, a machine learning method based on Convolutional neural networks, that learns the characteristics of G4 sequences and accurately predicts G4s outperforming state-of-the-art methods. We provide both a standalone implementation of the trained model, and a web application that can be used to evaluate sequences for their G4 potential.

## Introduction

**FIGURE 1:**
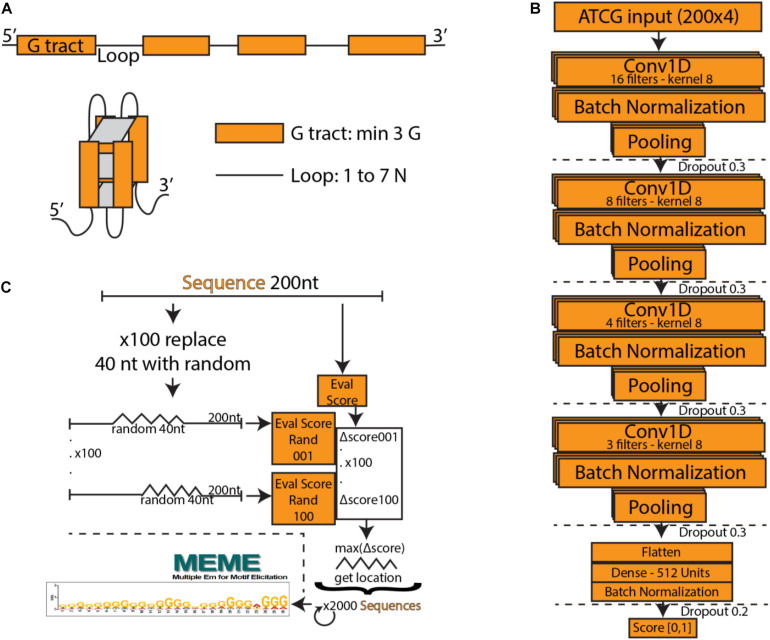
**(A)** Schematic of a typical G-quadruplex structure consisting of four G tracts with a minimum length of three, connected by non-specific loops. **(B)** PENGUINN convolutional neural network model. **(C)** Identification of G-quadruplex subsequences via randomized mutation.

G-quadruplexes (G4s) are stable secondary structures of nucleic acids that occur when quartets of guanines are stabilized by a monovalent cation (Gellert et al., 1962) and form a characteristic layered structure (Sen and Gilbert, 1988; Figure 1A). G4s are known to play important roles in several biological processes, such as DNA replication, damage response, RNA transcription and processing, transcriptional and translational regulation and others (Spiegel et al., 2020). Owing to their importance as modulators of genomic function, G4s have been studied extensively, and several attempts have been made to model their structure in a predictive manner and several experimental methods for their identification have been developed (Lombardi and Londoño-Vallejo, 2020).

Early methods of G4 prediction were focused on the identification of a consensus motif, using a regular expression matching approach, often complemented by involved scoring calculations. An example of such methods is Quadparser (Huppert, 2005). It was not until a high-throughput sequencing method for genome wide identification of G4s (G4-Seq) was established (Chambers et al., 2015) that we started understanding how common G4s were, and how hard it is to accurately predict their genomic location. Out of over 700 thousand G4s identified in the human genome by high-throughput sequencing, approximately 450 thousand were not predictable by computational methods at the time. The incentive for the improvement of G4 prediction computational methods and a dataset that would allow us to do so, became evident. A second wave of computational methods attempted to predict G4 locations after the publication of this dataset. Among the most accurate and still functional methods are G4Hunter (Bedrat et al., 2016), which expands the regular expression methods by taking into account G-richness and G-skewness, Pqsfinder, which focuses on allowing customization for non-canonical G4s and was trained on the G4-Seq dataset (Hon et al., 2017), as well as Quadron (Sahakyan et al., 2017), a Machine Learning (ML) method trained on the G4-Seq dataset recognizing canonical sequence motifs with 12-nt maximum loop size, utilizing the tree based Gradient Boosting Machine approach.

This second generation of G4 identification methods utilizes ML to classify sequences based on their G4 forming potential. Generally, ML describes the field of computer science that implements mathematical models which enable computers to learn concepts and patterns embedded in data. One of the largest subfields of ML deals with the development of artificial Neural Networks (NNs), which were initially proposed as simplified models of neuronal function (Fitch, 1944) and have recently revolutionized the fields of speech recognition and image classification (LeCun et al., 2015). The recent breakthrough in the field of NNs involves the utilization of Deep NNs consisting of a large number of neuronal layers. A specific subset of these Deep NNs uses a process known as convolution to learn increasingly complex representations of patterns in raw data. These NNs are called Convolutional Neural Networks (CNNs). An important characteristic of CNNs is their ability to operate on raw data such as images, time-series, DNA/RNA sequences, without the need for complicated feature extraction. The flipside of this ability is their need for large amounts of data. Coupled with the novel availability of high-throughput biological data (Emmert-Streib et al., 2020), Deep NNs are quickly becoming feasible in the field of bioinformatics (Tang et al., 2019). Another important current field of research is the interpretation of Deep NN models, which are often seen as “black boxes” due to their complexity. Convolutional Neural Networks for G4 prediction were implemented in the method G4detector (Barshai and Orenstein, 2019).

Here, we present PENGUINN, a CNN based approach for the identification of G4s from raw DNA sequence data, trained on G4-Seq high throughput human data. We establish that PENGUINN outperforms the state-of-the-art methods in a high background testing set that simulate high genomic variation, and interpret aspects of the learned model, validating its learning against known characteristics of G4 sequences. All data, training scheme, trained models, and functional code can be found at https://github.com/ML-Bioinfo-CEITEC/penguinn. An easy to use Web Application that can run the trained model for user submitted sequences in real time is also made available at https://ml-bioinfo-ceitec.github.io/penguinn/.

## Materials and Methods

### Training and Evaluation Datasets

Our dataset was generated from high-throughput sequencing of DNA G-quadruplexes from the human genome. We used genomic coordinates obtained from a G4-seq experiment (Chambers et al., 2015) (GEO: GSE63874). The coordinates were in three separate sets analogous to three different stabilizers – K^+^, PDS, and K^+^ together with PDS. We pooled all three datasets. Using the original bed files and the hg19 genome annotation we extracted DNA sequences using bedtools (Quinlan and Hall, 2010). We reverse complement all sequences mapping on the minus strand and merge them with sequences on the plus strand. All sequences which were longer than 200 nt were centered and cut to the length of 200 nt. Shorter sequences were randomly padded from both sides with Ns to become 200 nt long. We referred to this adjusted set as the positive set of the classification problem. For every sequence in our positive set we created a negative sequence of the same length from a random coordinate from hg19 that did not overlap with any of the coordinates from the positive set. Sequences shorter than 200 nt were again randomly padded with Ns. The sequences thus obtained formed a negative set.

We randomly selected 300 k sequences (150 k positive and 150 k negative) from the samples as a training set with pos:neg ratio 1 (the 1:1 dataset). We also randomly selected 300 k sequences to create the pos:neg training datasets of 1:9, 1:99, and 1:999, containing 30, 3 k, and 300 positives, respectively. These datasets correspond to 50%, 10%, 1% and 0.1% positive admixtures, respectively. The training datasets were split into training and evaluation groups, used for network optimization.

We selected four independent sets consisting of 100k sequences each as our final held-out evaluation sets, never seen during any training or hyperparameter selection step. The individual test sets have the same pos:neg ratio as the training sets – 1:1, 1:9, 1:99, and 1:999.

### Training Scheme

We utilized a Convolutional Neural Network consisting of four convolution layers with kernels of size 8 and 16, 8, 4, and 3 filters, respectively. The output of each convolutional layer goes through a batch normalization layer, max-pooling layer and dropout layer with the dropout rate 0.3. The output of the last layer is flattened and goes through a densely connected layer with ReLU activation function. The last layer is formed of a single neuron with a sigmoid activation function, which assigns to each input DNA sequence a probability of having a G4 structure. Our model was implemented in Python using the Keras library with Tensorflow backend. We used Adam optimizer with β_1_ = 0.9 and β_2_ = 0.99, the learning rate was set to 0.001. The loss function was binary crossentropy. The model was trained over 15 epochs, the chosen batch size was 32. Input to the neural network is a one-hot encoded DNA sequence. Figure 1B outlines the architecture of the network.

### Evaluation Scheme

We evaluated our model against five other state-of-the-art methods. First, we tested against the widely used regular expression “(G{3,}[ATGCN]{1,7}){3,}G{3,}” (four or more G stretches longer than 3 nucleotides, connected by 1-7 nucleotide stretches of any nucleotide). We implemented the regular expression in python, returning a boolean expression dependent on the presence of a match in the presented sequence. The remaining three methods that were developed for the scoring of G4 forming potential are G4Hunter (Bedrat et al., 2016), Quadron (Sahakyan et al., 2017) and Pqsfinder (Hon et al., 2017). For practical reasons we re-implemented G4Hunter in python (code available at our repository). We used a window of size 25 nucleotides as proposed in the original paper, and a score threshold 0 to see all putative G4s. For every input sequence, the output of our implementation is the highest score of all subsequences. If no G4 has been found, the output score is 0. We ran Quadron with the default parameters. For every input sequence, we considered only scores assigned to the plus strand and we took the maximum of all scored G4s present in the sequence. If no score has been assigned, the output score was zero. For testing Pqsfinder was used following command: “pqsfinder (sequence, strand = ‘+’, overlapping = TRUE, verbose = FALSE),” as an output we took the highest scoring G4, if none has been found, the score was set to 0. Lastly, we compared our model to another ML model G4detector (Barshai and Orenstein, 2019). We ran it in the testing mode using three available models trained on random negatives and positives with K^+^, PDS, and K^+^ + PDS stabilizers.

### Evaluation on Independent Datasets

For additional testing we created two independent datasets. The first dataset was generated from a recent publication (Marsico et al., 2019) which was published by the same research group from which the training dataset was obtained. The group has improved the G-quadruplex sequencing method and provides new whole genome G4 map for human. We used generated coordinates (GSM3003539 and GSM3003540) with G4 structures from two different stabilizers – K^+^ and PDS. From the obtained data, positive and negative set was generated the same way as the training and evaluation datasets (see section “Training and Evaluation Datasets”). From the positive and negative set we randomly selected sequences to get four datasets consisting of 100 k sequences each. The individual sets have the same pos:neg ratio as the training and evaluation sets – 1:1, 1:9, 1:99, and 1:999. We will refer to these datasets as Marsico dataset.

The second dataset was obtained by a G4 ChIP-seq approach (Hänsel-Hertsch et al., 2016) and contained G-quadruplexes from *in vivo* data. We used genomic coordinates generated in this experiment (GSE76688). We merged all the obtained coordinates from different methods and cell lines into one file and using bedtools and hg19 genome extracted corresponding genome sequences. The rest of the processing steps was identical to the steps described in “Training and Evaluation Datasets” section. From the generated positive and negative set we randomly selected sequences to get four datasets containing the same number and ratio of pos:neg sequences as in the Marsico dataset. We will refer to these created datasets as Hänsel-Hertsch dataset.

PENGUINN and all other tools described in “Evaluation Scheme” section were evaluated on the two independent datasets. The parameters of the other state-of-the-art methods were used the same way as described above.

### Training and Evaluation on Non-Human Datasets

To show PENGUINNs ability to work with different type of G4 sequences, we trained a model only on mouse G-quadruplexes. We obtained data from *in vitro* G4 map of mouse genome (Marsico et al., 2019) with genomic coordinates (GSM3003547 and GSM3003548) under K^+^ and PDS stabilizers. We prepared both training and testing datasets the same way as human training and testing datasets, but using GRCm38 mouse genome instead of human genome (see section “Training and Evaluation Datasets”). The same model training scheme as for human G4s was used (see section “Training Scheme”).

### Interpretation of Model

In order to interpret the model, we have attempted to isolate the particular features detected by the model which weigh the most for its decision making. For each positive testing sample we have produced 100 sequences of the same length containing a random stretch of 40 nucleotides for every possible position. For each such sequence we re-evaluate and calculate the average degree of score change for the subsequence. The subsequence that produces the largest drop is marked as the “most important” and extracted (Figure 1C).

### Code Availability and Web Application

PENGUINN was developed in Python. All code accompanied by the trained models, all training data and the installation instructions can be found at https://github.com/ML-Bioinfo-CEITEC/penguinn. Moreover, we have converted the trained PENGUINN Keras model into TensorflowJS and developed a simple web application, available at https://ml-bioinfo-ceitec.github.io/penguinn/. The web application code can be found in the gh-pages branch of the PENGUINN GitHub repository.

## Results

### Selection of Training Ratio

Scanning across genomic regions for a specific and relatively rare structural element is a task that involves a heavy class imbalance, since the background sequence will heavily outnumber the target element by orders of magnitude. It is hard to know the exact prevalence of G4s in the genome or at least the areas of the genome one would scan, but the more imbalance datasets should approximate a realistic ratio more closely. A rough estimate could come from equally dividing the approximately 700 thousand known G4s over the 6 billion bases of the human genome, giving us an approximate ratio of a G4 located every 8 thousand base pairs. For this reason, we have produced four datasets with increasing positive to negative ratio (pos:neg) by one order of magnitude each time. Starting from the highly unrealistic 1:1 dataset with equally balanced classes (50%) and then going up to 1:9 (10%), 1:99 (1%), and 1:999 (0.1%) ratio datasets. We have also acquired a dataset (Lombardi and Londoño-Vallejo, 2020) with high class imbalance in the opposite direction consisting of 298 positives and 94 negatives (3:1 dataset).

Initially, we explored the possibility of training models in equally imbalanced datasets and then using them to improve prediction accuracy. However, we could not see any measurable improvement for training with a matching pos:neg mixture when considering the area under the precision sensitivity curve for our models, or when using an iterative negative selection technique that previously showed improvement in a different genomic classification task (Georgakilas et al., 2020; Supplementary Figure 1). Since there does not appear to exist a major difference in performance between these models, we have elected to use the model trained on 1:1 as our main trained model. Plotting the F1 score against the prediction score of our method for each testing dataset (Supplementary Figure 2) we have identified two score values that we proposed as score thresholds for our method (precise: 0.85, sensitive: 0.5). Users are allowed to set their own cut-off threshold for their results depending on their needs, but having proposed score thresholds helps new users guide their decisions to more meaningful thresholds. For clarity of presentation, on all evaluations against state-of-the-art methods we will designate these thresholds as PENGUINN(s) for the sensitive, and PENGUINN(p) for the precise threshold.

### Comparison to Regular Expression

**FIGURE 2:**
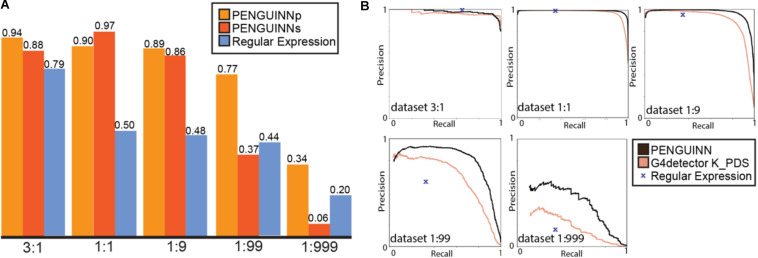
**(A)** F1 score for PENGUINNp (precise), PENGUINNs (sensitive) and Regular Expression with datasets of different pos:neg ratio. **(B)** Precision-Recall curve comparison of PENGUINN and best performing state-of-the-art method G4detector K_PDS and Regular Expression in datasets of different pos:neg ratio.

A commonly used method for G4 identification is the use of a sequence pattern, also called a regular expression, consisting of up to four stretches of Gs with a minimum length of three, spaced by random nucleotide sequences with a maximum length of seven (for exact expression see Materials and Methods). This method was first proposed over 15 years ago (Huppert, 2005), and a simplified version “(G{3,}[ATGCN]{1,7}){3,}G{3,}” (four or more G stretches longer than 3 nucleotides, connected by 1-7 nucleotide stretches of any nucleotide) has been commonly used since then. We have directly compared PENGUINN to this regular expression in all our testing datasets. Since the regular expression cannot return a score and will only produce a binary result, it is not possible to produce a ROC curve or similar metric across scores. Our models outperformed the G4 regular expression in all datasets with increasing difference as datasets became more negative heavy (Table 1 and Figure 2). Despite being a widely used way of identification for G4s, the regular expression lacks a scoring system to prioritize sequences compared to more elaborate methods such as PENGUINN.

**TABLE 1:** Precision and recall values for static score prediction of regular expression, PENGUINNs (sensitive), and PENGUINNp (precise) on a scale of imbalanced datasets.

**Dataset**	**3: 1**		**1: 1**		**1: 9**		**1: 99**		**1: 999**	
**Tool**	**Precision**	**Recall**	**Precision**	**Recall**	**Precision**	**Recall**	**Precision**	**Recall**	**Precision**	**Recall**
Regular expression	0.995	0.657	0.993	0.335	0.952	0.323	0. 606	0. 349	0.145	0.330
PENGUINNs	0.780	1. 000	0. 967	0. 964	0. 778	0. 963	0.231	0.955	0.030	0.940
PENGUINNp	0.952	0. 926	0.996	0. 818	0. 973	0. 823	0. 733	0. 814	0. 214	0. 790

*Dataset 3:1 contains 298 positives and 94 negatives, datasets 1:1, 1:9, 1:99, and 1:999 have a total of 100,000 left out examples each with the denoted pos:neg ratio.*

### Comparison to State-of-the-Art Methods

We proceeded to evaluate our method against four other state-of-the-art methods on the same benchmark datasets. We compared their performance across the whole range of prediction scores using the precision-recall area under curve for each evaluation dataset. There is an evident trend of quickly diminishing performance as datasets become more realistic in ratios with more negatives. Our method also loses performance under these circumstances, but at a much slower rate, pointing at a comparative improvement when used for realistic highly imbalanced datasets (Table 2). We have selected the best performing state-of-the-art method for direct comparison using detailed precision-recall curves for each dataset (Figure 2B). It becomes evident that as the class imbalance increases, both methods lose performance, but PENGUINN manages to retain a higher level of precision/sensitivity even at highly imbalanced datasets. Comparison with all other state-of-the-art programs shows similar patterns as ratios become more realistic (Supplementary Figure 3A).

**TABLE 2:** Area under the precision-recall curve for PENGUINN and 4 state-of-the-art programs.

**Dataset**	**3:1**	**1:1**	**1:9**	**1:99**	**1:999**
PENGUINN	0.978	0.994	0.966	0.796	0.402
G4detector K PDS	0.965	0.979	0.906	0.637	0.188
G4detector PDS	0.937	0.978	0.899	0.585	0.152
G4detector K	0.941	0.978	0.888	0.552	0.124
G4Hunter	0.972	0.964	0.851	0.503	0.093
Quadron	0.965	0.828	0.671	0.522	0.150
PQSfinder	0.977	0.948	0.861	0.551	0.101

*Underlined are the best performances for each evaluation dataset. Dataset 3:1 contains 298 positives and 94 negatives, datasets 1:1, 1:9, 1:99, and 1:999 have a total of 100,000 left out examples each with the denoted pos:neg ratio.*

To ensure that our method was not overfitted on the dataset used for training, we decided to evaluate our method, as well as other state-of-the-art programs against two independent datasets (Hänsel-Hertsch et al., 2016; Marsico et al., 2019). On the Marsico dataset, all programs showed similar results as on the original testing dataset (Supplementary Figure 3B). On the Hansel-Hertsch dataset, all programs performed poorly compared to their performance on our original dataset. Despite this fact, PENGUINN outperformed all other state-of-the-art programs even on this dataset (Supplementary Figure 3C). This dataset is not easily comparable with naked DNA G4-seq as it is dependent on a cell line in which many G4s are tightly packed in nucleosomes. We do not suggest that absolute performance metrics in this cell line are representative of the real power of each prediction method, but his result reinforces our trust that PENGUINN’s performance is not the result of overfitting on the training dataset.

### Training on Non-Human Datasets and Cross-Species Comparisons

Since the Marsico dataset (Marsico et al., 2019) contained information about the genomic locations of G4s on various species, we decided to evaluate our model on a possible cross-species comparison, and also to train a model on other species data. For this task, we selected the mouse dataset since it had the largest number of peaks, adequate for training a model. We then proceeded to evaluate and compare the two models (Trained on human and Trained on mouse) against left out datasets from each of the two species. As expected, the model trained on human performed slightly better on the human dataset, and the model trained on mouse better on the mouse evaluation dataset (Table 3 and Supplementary Figure 4). We also evaluated the human trained model against two evolutionary distant organisms, the nematode *Cenorhabditis elegans*, and the plant *Arabidopsis thaliana*. These datasets contained a much smaller number of peaks that were not enough for training their own independent models. The human trained model can predict G4s in these species, but the performance of the model is as expected lower than what it is for human or mouse (Supplementary Figure 5).

**TABLE 3:** Area under the precision-recall curve for PENGUINN models trained on human and mouse datasets, and evaluated on varying pos:neg ratios of human and mouse G4s.

**Dataset/model**	**Trained on human**	**Trained on mouse**
Dataset 1:1 human	0.994	0.987
Dataset 1:9 human	0.966	0.931
Dataset 1:99 human	0.796	0.718
Dataset 1:999 human	0.402	0.373
Dataset 1:1 mouse	0.986	0.992
Dataset 1:9 mouse	0.912	0.945
Dataset 1:99 mouse	0.658	0.795
Dataset 1:999 mouse	0.236	0.361

*Underlined are the higher values for each evaluation dataset.*

### Interpretation of PENGUINN Model

Deep Learning models consist of complex networks of neurons that abstract information in hard-to-interpret ways. Often these models are considered uninterpretable black boxes. However, this can lead to pitfalls such as learning data artifacts instead of real signal. To control against such pitfalls, we identified what our model considered the most important 40 nucleotide subsequence for 2000 samples, using a randomized permutation approach. We sorted by the degree of change when randomized and used the top 2000 of these sequences to identify enriched motifs using Multiple Em for Motif Elicitation (MEME) (Bailey et al., 2006). The top motif extracted (Figure 1C) is indeed a motif containing several G-tracts which confirms the known theory of G4 formation and demonstrates that our model did not primarily learn some artifact such as padding length. The motif produced by MEME is an aggregate of several similar motifs found in these sequences, and as such is not expected to appear as an exact “consensus” G4.

We proceeded to evaluate negative examples in the same way, attempting to identify possible motifs that could weigh the classification model toward the negative class. Using 2000 such sequences, we identified some motifs with high *E*-value that do not show similarity among them or with the positive motifs. Even the most common of these motifs was only found in 40 out of 2000 sequences, while the G-rich motif in the positive samples was found in 1961 out of 2000 sequences. These results show us that even though there may be some motif overrepresentation in our negative data, there does not appear to exist a definitive motif that could be biasing our model.

We intersected PENGUINN predictions at a 0.5 score threshold against the predictions of G4detector and the regular expression method. PENGUINN identified 3818 “unique” G4s, against 440 of G4detector, and 24 of the regular expression. PENGUINN and G4detector had 27556 sequences in common which could not identified by regular expression alone (Supplementary Figure 6A). We proceeded with a motif finding analysis using a randomized permutation approach on the sequences that were correctly identified only by PENGUINN. To look for sequences beyond the G-rich primary sequence, we allowed up to three top subsequences per sample to be considered. In total, we produced 2100 important sub-sequences, out of which 1281 contained a roughly T/C rich motif sequence (Supplementary Figure 6B).

### Web Application

Paradoxically, although elaborate ML models, such as PENGUINN, vastly outperform the simple regular expression search for G4s, its use persists to date. Beyond the familiarity of the method, we believe that any technical obstacle, however trivial, will deter non-technical users from using other methods. As such, we decided to create a straightforward web application that uses our best trained model to evaluate user submitted sequences in real time. The web application can be found here: https://ml-bioinfo-ceitec.github.io/penguinn/. The user can input a single sequence, a fasta formatted input, or several sequences in multiple lines. The sequences will be evaluated, and a score along with threshold evaluation returned.

## Discussion

In this study we present PENGUINN, a convolutional neural network based method that outperforms state-of-the-art methods in the identification of nuclear G4s in highly imbalanced datasets. PENGUINN is more robust than other methods when the pos:neg ratio increases by several orders of magnitude. However, there is still space for improvement in the prediction. We believe that a more elaborate modeling of the real variation of the background genome could benefit predictive methods of this type. Such undertaking is beyond the scope of this study.

Beyond the development of a highly effective predictive model, we have explored the interpretation of what the model has learned. As expected, the model identified regions of high G content as better potential targets, and has scored very highly regions showing periodic G stretches, a structural feature known to define G4s. Convolutional Neural Networks are notorious for being hard to interpret, as deeper network layers further abstract information from the first layers. We believe that interpreting the network to the extent that we can conceptualize the type of sequences it has learned to identify is an important step for genomic sequence deep learning studies.

To allow for easier adoption of our method, we have developed both a standalone version and a web application that can be used without any knowledge of programming. The repository https://github.com/ML-Bioinfo-CEITEC/penguinn contains all models, data, and thorough installation and usage tutorials. The web application can accept sequences ranging from 20 nt up to hundreds of nts. For sequences smaller than 200 nt, our method will pad the sequence with Ns randomly on each side. This may create a variation in scores for really short input sequences. For sequences larger than 200 nt, our method will extract 200 nt around the midpoint of the sequence. This means that the whole sequence is not evaluated but just the middle 200 nt. Users should attempt to preprocess their data as much as possible, centering their potential G4 sequence.

In conclusion, PENGUINN is a powerful method, based on cutting edge Deep Learning architecture, that increasingly outperforms the state-of-the-art methods in classifying G4s in more realistic highly imbalanced datasets. Despite the sophistication of the method, we have developed a simple web application to assist users coming from non-bioinformatic backgrounds to use the method. We also provide all training and testing datasets in an effort to empower researchers to produce better, more accurate methods for realistic highly imbalanced datasets.

## Data Availability Statement

All datasets presented in this study are available at https://github.com/ML-Bioinfo-CEITEC/penguinn.

## Author Contributions

PA and EK designed the study. PA had the oversight of the study. EK and JP developed the machine learning method. PS developed the web application. PA, EK, JP, and PS wrote the manuscript. All authors contributed to the article and approved the submitted version.

## Conflict of Interest

The authors declare that the research was conducted in the absence of any commercial or financial relationships that could be construed as a potential conflict of interest.
