# Supplementary figures and images for: PENGUINN: Precise Exploration of Nuclear G-Quadruplexes Using Interpretable Neural Networks
Source: Front Genet. 2020 Oct 27;11:568546. doi: 10.3389/fgene.2020.568546 (PMC7653191; doi:10.3389/fgene.2020.568546)

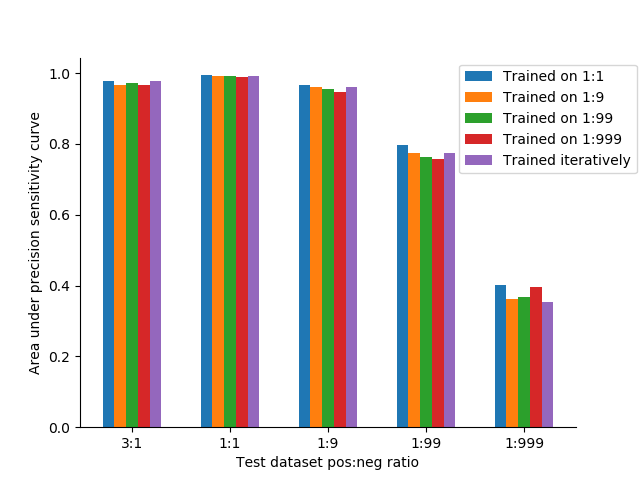

Supplement: Supplementary file 1 [file Image_1.PNG]

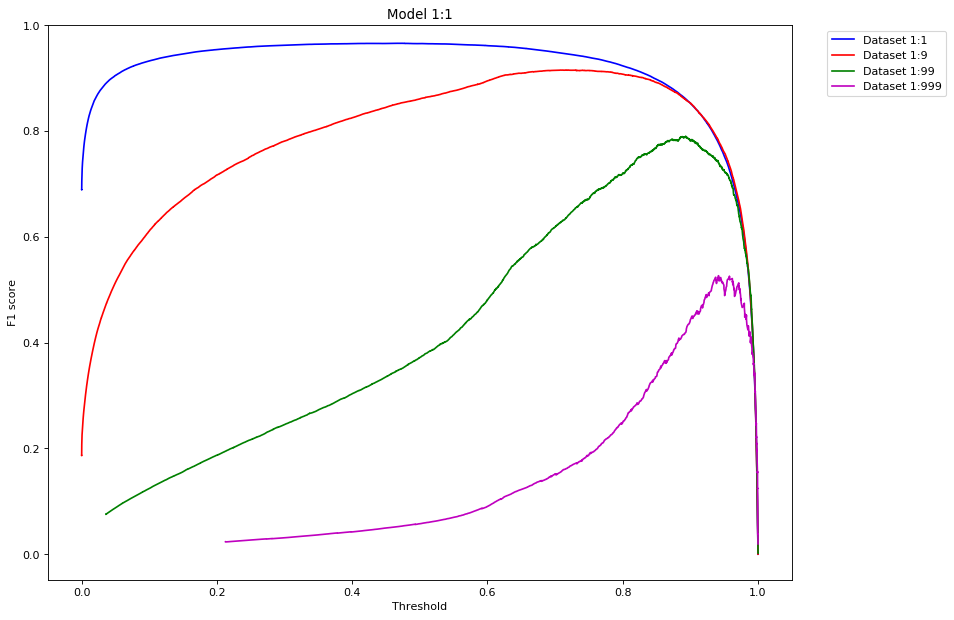

Supplement: Supplementary file 2 [file Image_2.PNG]

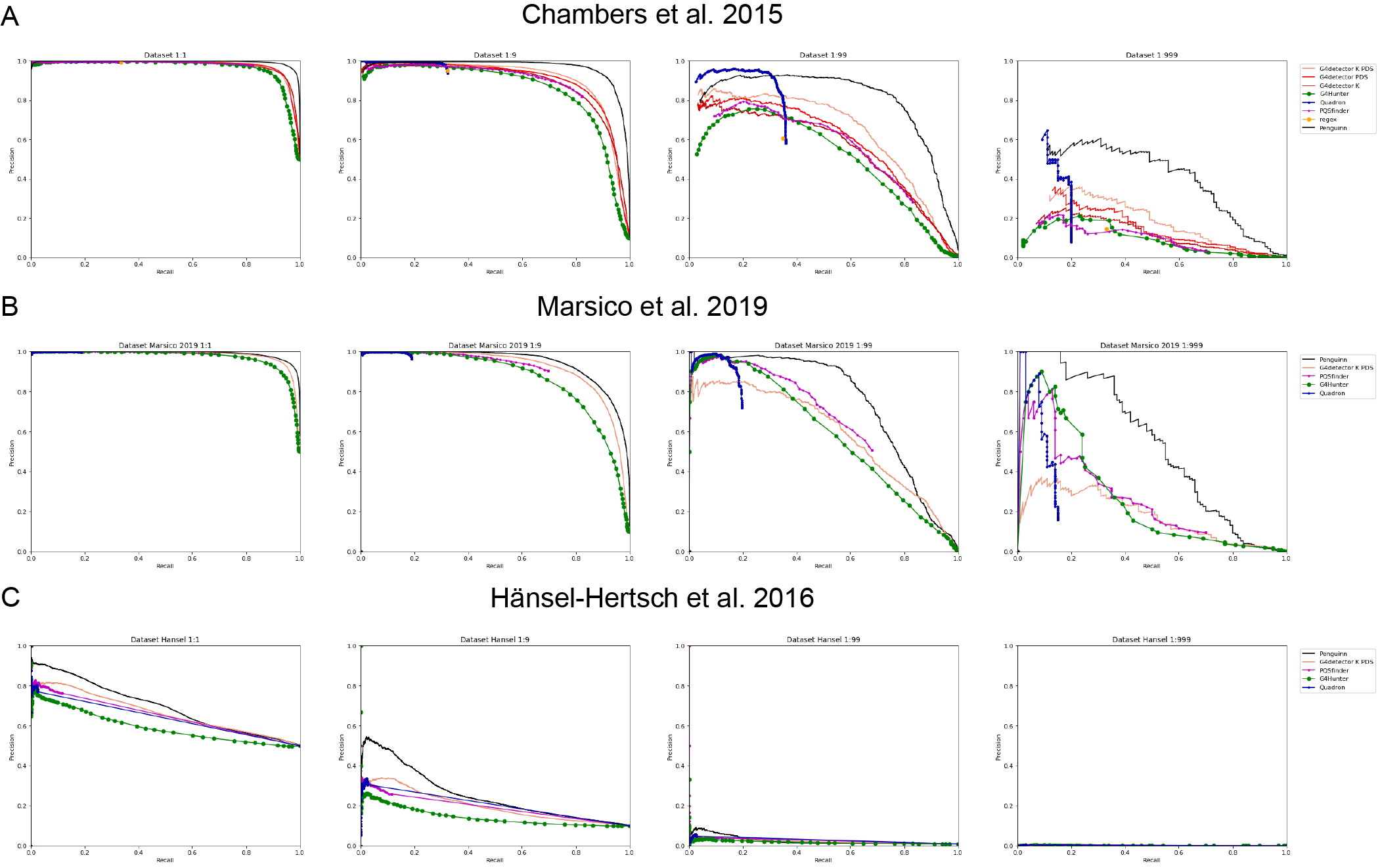

Supplement: Supplementary file 3 [file Image_3.PNG]

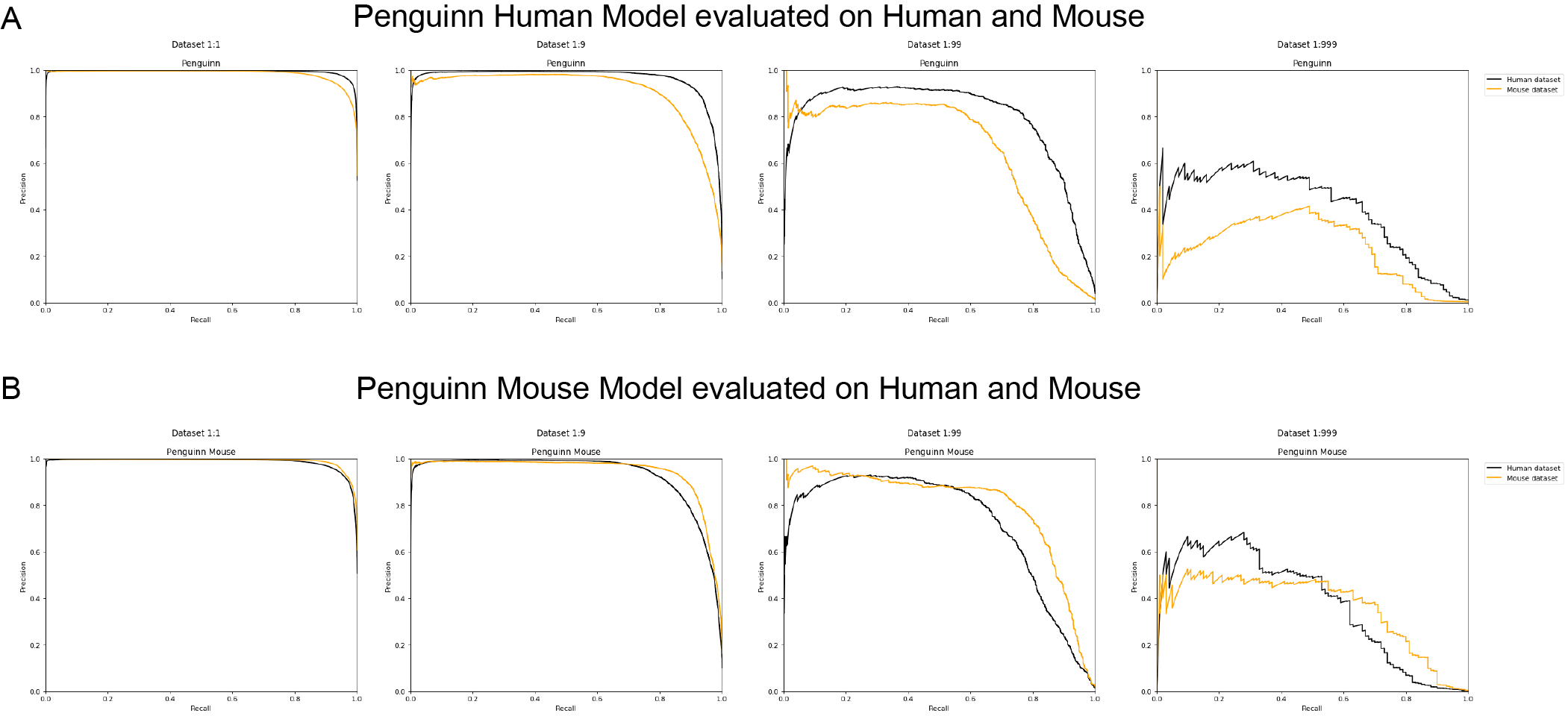

Supplement: Supplementary file 4 [file Image_4.PNG]

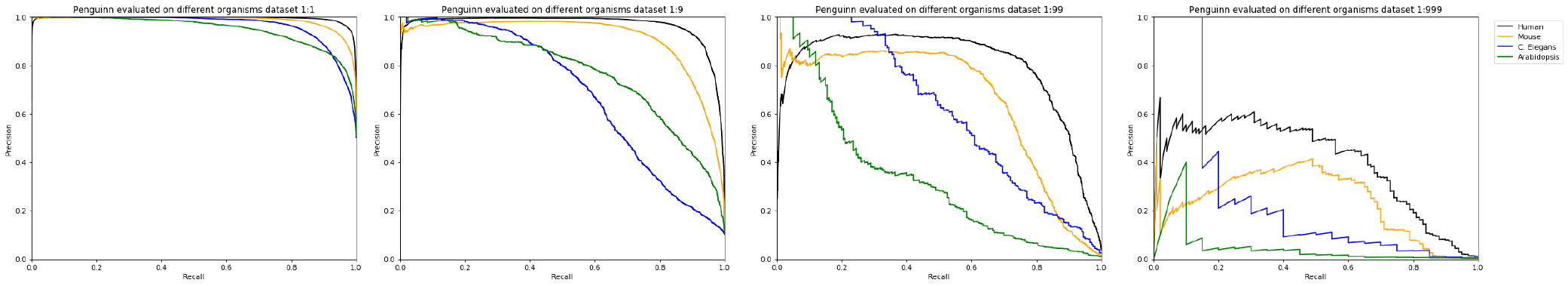

Supplement: Supplementary file 5 [file Image_5.PNG]

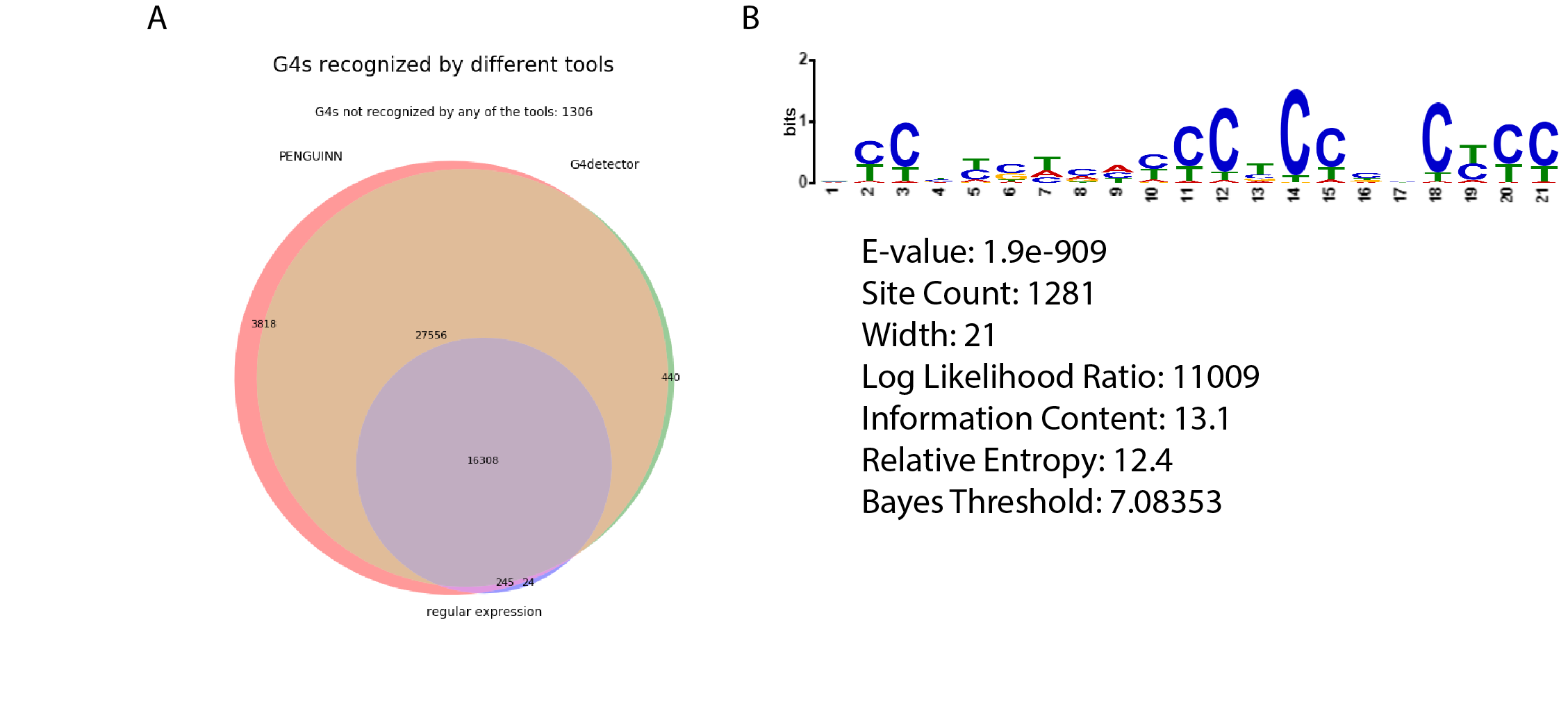

Supplement: Supplementary file 6 [file Image_6.PNG]
